# Squamous cell carcinoma arising in an epidermal cyst of urinary bladder associated with vesicolithiasis: A case report and review of the literature

**DOI:** 10.1016/j.ijscr.2021.106290

**Published:** 2021-08-06

**Authors:** Mujtaba Haidari, Ramin Saadaat, Haider Ali Malakzai, Jamshid Abdul-Ghafar

**Affiliations:** Department of Pathology and Clinical Laboratory, French Medical Institute for Mothers and Children (FMIC), Kabul, Afghanistan

**Keywords:** EC, epidermal cyst, MT, malignant transformation, UB, urinary bladder, SqCC, squamous cell carcinoma, Squamous cell carcinoma, Urinary bladder, Malignant transformation, Case report

## Abstract

**Introduction and importance:**

Epidermal cysts can rarely occur in internal organs, but epidermal cyst of the urinary bladder is extremely rare with a very low tendency for malignant transformation. This article will guide the physicians to be aware of such rare complex neoplastic combinations to take proper decisions in management and treatment of the patients.

**Case presentation:**

A 40-year-old rural male patient with hematuria and urinary frequency presented to a hospital. Ultrasonography reported a cyst and a 2 × 1.5 cm stone in the urinary bladder. Surgery planned to remove the cyst and the stone. During the surgery, a cyst with solid parts and a stone were observed. The cyst had superficial penetrations into the bladder wall, the patient refused to done radical cystectomy, therefore the cyst excised from the bladder and sent for histopathological examination. The histopathological examination confirmed a well differentiated invasive squamous cell carcinoma arising from epidermal cyst with detrusor muscle invasion, staged pT2aNx. After diagnosis the patient received chemotherapy and improved his urinary symptoms. The possibility of further radical cystectomy and radiotherapy in neighboring countries remains open.

**Clinical discussion:**

Urinary bladder cysts associated with stones are uncommon but the occurrence of epidermal cyst in the urinary bladder is an extremely unusual incidence and malignant transformation of this lesion made it even rarer.

**Conclusion:**

Despite the rarity of malignant transformation in epidermal cyst and the unknown etiology of its occurrence in the urinary bladder, further clinicopathological and molecular studies are needed to reveal the possible pathogenesis with involved risk factors.

## Highlights

• Malignant growth must be considered in a chronic epidermal cyst that displays unusual features.
• Epidermal cysts can rarely occur in internal organs.
• This is the first case of epidermoid cyst in urinary bladder with malignant transformation.
• Both epidermal cyst malignancy and squamous cell carcinoma in the urinary bladder are more common in males than females.

## 1 Introduction

Epidermal cyst (EC), also known as sebaceous cyst, is the most common cyst of the skin [1], which is characterized by keratinized stratified squamous epithelium with distinct granular layer producing lamellated keratin and sebaceous material without calcification [2], [3]. ECs are more frequent in males than females [4] and can occur at any age and any part of the body, however, they are usually present in the hair-bearing areas such as the scalp, face, neck, trunk, and back regions [3], [4], [5]. Although cases of ECs are reported in the urinary tract involving the kidney [6] and penis, its occurrence is extremely rare in urinary bladder and ureter (Table 1). ECs can rarely transform into malignant neoplasms such as squamous cell carcinoma (SqCC) and basal cell carcinoma. According to the literature, the most frequent sites for ECs transforming into SqCCs are head and neck [1]. Here we present probably the first case of an EC arising in the urinary bladder (UB) which is associated with vesicolithiasis and malignant transformation (MT) into SqCC. The work has been reported in line with the SCARE 2020 criteria [7].

**Table 1:** Ureter and urinary bladder epidermoid cysts with or without malignant transformation reported in the literature.

Author, year	Age/Sex	Clinical presentation	Method of diagnosis/treatment	Location	Biopsy	Pathology	Malignant transformation	Follow up
Ishizaki, et al., 2003 [5]	72/M	Right flank pain with history of renal stone since 4 years	Sonography: right hydronephrosis Intravenous urography: right kidney doesn't visualized Retrograde pyelography: showed complete obstruction at the right ureter. Computed tomography and Magnetic resonance imaging: small calcified mass with partial enhancement at the same location as well as right hydronephrosis	Upper part of ureter	Yes	Epidermoid cyst	No	Not applicable
Wang, et al., 2013 [14]	31/M	Non	Sonography: Avascular lesion with onion-ring hyper and hypoechoic pattern. Computed tomography: cystic mass at right wall of the urinary bladder	Right lateral wall	Yes	Epidermal cyst	No	After 18 months no recurrence noted
Akyuz, et al., 2019 [4]	59/F	Burning pain during urination	Sonography: three polypoidal lesions the largest being 6 mm in diameter	Left lateral wall	Yes	Epidermoid cyst	No	After 3 months no recurrence noted
Puppala, et al., 2019 [6]	45/F	Lower abdominal pain, urinary frequency, and left flank pain	Sonography: focal soft tissue thickening with calcified speck in the posterior wall of the urinary bladder. Computed tomography: focal nodular thickening of posterior wall of the urinary bladder with calcification Magnetic resonance imaging: a well-defined lesion in the posterior wall of the urinary bladder	Posterior wall	Yes	Epidermoid cyst	No	Not applicable

## 2 Presentation of case

**Fig. 1:**
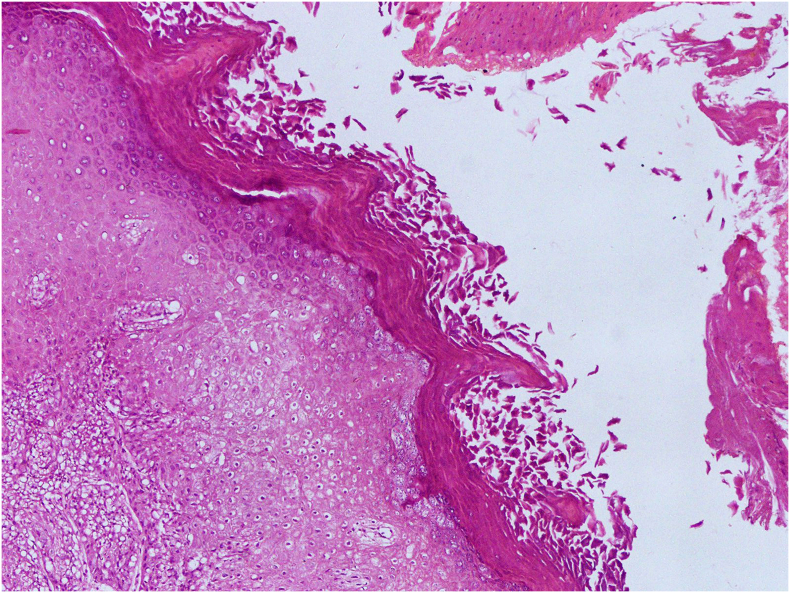
Cystic lesion lined by stratified squamous epithelium showing prominent granular layer with variable amounts of keratinous debris and some intervening fragments of packed keratin lamellae.

**Fig. 2:**
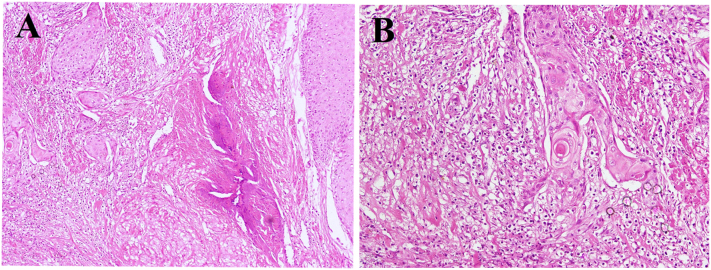
Malignant squamous cell carcinoma arising from the epidermal cyst (A) low-power view showed invasion of the neoplastic cells into bladder wall with desmoplastic stroma. (B) High power view showing the definite squamous cell carcinoma along with keratin pearl formation.

A 40-year-old peasant and rural resident man from a low-income family presented to a hospital, complaining from hematuria and urinary frequency for 5 months. The patient is a tobacco addict with no other systemic diseases or family history for malignancy. On physical examination, no other visible abnormality was noted. Ultrasonography reported a cyst with solid areas and a 2 × 1.5 cm stone (vesicolithiasis) in the urinary bladder. Laboratory examinations showed gross hematuria and pyuria. Hemoglobin, kidney function test and liver function test were in normal ranges. Surgery was planned to remove the stone (vesicolithiatomy) and the cyst. During the surgery, a cystic lesion and a stone were observed. The cyst also had solid areas and during resection it showed superficial penetrations into the bladder wall. The cyst was excised and send for histopathological examination. Grossly it was consisting of fragmented gray-white cyst wall tissue, with the largest tissue fragment measures 3 cm in its greatest dimension. The inner surface of cyst wall fragments was tan and flaky in appearance. The entire tissue specimen was submitted for microscopic evaluation. Histologically features showed detrusor muscle of the urinary bladder with associated epidermal cyst lined by keratinized stratified squamous epithelium exhibiting keratin flakes and prominent granular layer (Fig. 1). The cyst revealed focal transformation to SqCC characterized by sheets of polygonal squamous cells connected with each other by intercellular bridges having abundant eosinophilic cytoplasm, hyperchromatic nuclei, and visible nucleoli with increased number of mitotic figures (Fig. 2A). Keratin pearls formation and invasion into the detrusor muscle of the urinary bladder were also observed (Fig. 2B). The diagnosis of well differentiated invasive SqCC arising in epidermal cyst with superficial involvement of muscularis propria was made. The tumor was reported stage-II as per American Joint Committee on Cancer (AJCC) pTNM classification, 8th edition. Since a standard oncology center with radiotherapy facilities is not available in the country, the patient went to the neighboring country (Pakistan) for further treatment and follow-up. According to the patient statement he has received a course of chemotherapy. After the chemotherapy patient relatively felt better and stopped further treatment. Due to his poor economic situation, he has refused further surgery (complete cystectomy) as suggested by his physicians. He is alive after one year of follow-up and did not perform any further radiological or surgical procedures but the possibility of further radical cystectomy and radiotherapy in neighboring countries remains open.

## 3 Discussion

Ewing in 1942, proposed that epidermal remnants derived from Wolffian ducts in internal organs can be the possible source of EC development [5]. Although the presence of ECs in the skin usually show no symptoms, unless, it raptures, infected, or significantly increased in size, but the involvement of the other organs by ECs is often associated with symptoms specific to the involved organ [9]. The diagnosis of EC in solid organs and urinary tract can be presumptive and difficult to be correctly diagnosed. The presence of keratinized material in the urine sample suggests an epidermoid cyst in the urinary tract [5]. ECs are usually benign, however, a few cases with MT have been reported in the literature with a reported rate up to 0.045% [1], [8], [9]. SqCC arising in an EC is rarely suspected in routine clinical practice. ECs are regularly and often diagnosed by microscopic examination after excision [10].

Although the etiology of EC transforming into malignancy is unclear, tobacco use, male gender, chronic irritation, trauma and recurrent infection are the triggering factors that have been suggested. Persistent chronic irritation due to chronic inflammation or repeated trauma cause the damage to the lining cells of cyst and the repeated damage and repair process finally could result in dysplastic changes and invasive carcinoma [3], [9], [11]. In a long-standing EC, the MT can occur suddenly with some abnormal changes in the cyst including; a firm mass replacing the cyst, increase in size, discharge, pain, ulceration and bleeding inside the cyst which do not response to conservative treatment [7], [10], [12]. In a series of 94 cases of SqCC arising in EC reported, the most common site were head and neck (55%) followed by lower limb (13%), trunk (13%), perineum (8%) and upper limb (6%) with a slightly males' predominance (65%) [1]. Another review of 19 cases of SqCC arising in EC showed that majority of those cases were in males and the age ranged from 20 to 80 years old with mean age of 43.2 years [13]. However, previous reports of three cases of EC originating form urinary bladder revealed no malignant transformation (Table 1) but chance of malignant transformation in ECs are always possible. Age of our patient was 40-year-old and uniquely occurred in urinary bladder. Other than skin, malignancy arising from EC also occur in internal organs such as kidney, ureters, testes, intra-cranial regions and ovaries with extremely low occurrence rate [1]. A proper treatment for EC with MT is surgical excision, however, metastasis or local invasion of the tumor will demand additional therapeutic protocols including; disease palliative systemic chemotherapy or immunotherapy with PD-1 blockade using cemiplimab [12].

In our case, the middle-aged male patient presented with hematuria and urinary frequency has gone through vesicolithiatomy with removal of the cyst as primary therapeutic interventions. Considering the unknown etiology of EC transforming into malignancy, further clinicopathological and molecular studies are needed to identify the possible pathogenesis with involved risk factors.

## 4 Conclusion

EC is rare in internal organs and MT within the cyst is much rarer. In the current case, male gender and chronic irritation of the EC because of bladder stone possibly leads to MT. Malignant growth must be considered in a chronic EC that displays unusual features.

## Consent

Written informed consent was obtained from the patient for publication of this case report and any accompanying images. A copy of the written consent is available for review by the Editor-in-Chief of this journal on request.

## Sources of funding

None declared.

## Ethical approval

Ethical approval was obtained from the Research Ethics Committee of the concerned hospital.

## Author contribution

JAG and MH diagnosed the case. MH conceived the idea. MH and RS were the major contributors to the writing of the first draft of manuscript. JAG and HAM were major contributors to the critically revising of the manuscript and important intellectual content. All authors read and approved the final manuscript.

## Registration of research studies

Not applicable.

## Guarantor

Jamshid Abdul-Ghafar, MD, PhD.

## Provenance and peer review

Not commissioned, externally peer-reviewed.

## Declaration of competing interest

The authors report no declarations of interest.
